# Modeling advanced air mobility aircraft in data-driven reduced order realistic urban winds

**DOI:** 10.1038/s41598-023-50719-8

**Published:** 2024-01-03

**Authors:** Rohit K. S. S. Vuppala, Zack Krawczyk, Ryan Paul, Kursat Kara

**Affiliations:** 1 School of Mechanical and Aerospace Engineering, Oklahoma State University, Stillwater, Oklahoma USA; 2 School of Mechanical and Aerospace Engineering, Oklahoma State University, Stillwater, Oklahoma USA

**Keywords:** Aerospace engineering, Computational science, Fluid dynamics

## Abstract

The concept of Advanced Air Mobility involves utilizing cutting-edge transportation platforms to transport passengers and cargo efficiently over short distances in urban and suburban areas. However, using simplified atmospheric models for aircraft simulations can prove insufficient for modeling large disturbances impacting low-altitude flight regimes. Due to the complexities of operating in urban environments, realistic wind modeling is necessary to ensure trajectory planning and control design can maintain high levels of safety. In this study, we simulate the dynamic response of a representative advanced air mobility platform operating in wing-borne flight through an urban wind field generated using Large Eddy Simulations (LES) and a wind field created using reduced-order models based on full-order computational solutions. Our findings show that the longitudinal response of the aircraft was not greatly affected by the fidelity of the LES models or if the spatial variation was considered while evaluating the full-order wind model. This is encouraging as it indicates that the full LES generation of the wind field may not be necessary, which decreases the complexity and time needed in this analysis. Differences are present when comparing the lateral response, owing to the differences in the asymmetric loading of the planform in the full and reduced order models. These differences seen in the lateral responses are expected to increase for planforms with smaller wing loadings, which could pose challenges. Additionally, the response of the aircraft to the mean wind field, the temporal average of the full order model, was misrepresentative in the longitudinal response and greatly under-predicted control surface activity, particularly in the lateral response.

## Introduction

The aviation industry is undergoing a groundbreaking revolution with the emergence and deployment of a variety of vehicles falling under the Advanced Air Mobility (AAM) category^1^. AAM encompasses a diverse array of vehicle types, ranging from modern electric or hybrid-electric multi-rotor platforms designed to transport passengers and cargo to unmanned aircraft systems (UAS) serving the public in capacities such as delivery^2^, surveillance^3^, photography^4^, surveying^5^, and other applications^6^. The majority of proposed AAM operations are centered in urban areas, where high population densities and the public’s demand for swift delivery and transportation make flight operations economically viable. Given the numerous operations planned for densely populated urban environments, achieving rigorous safety standards are imperative for regulatory approval and public acceptance of this new category of flight operations.

Modern air vehicle engineering practice makes heavy use of simulation with gusts and turbulence present to assess stability, control, and robustness characteristics for new platforms as a new design progresses toward type certification^7^. One significant departure from existing operational models that provide frequent point-to-point transportation services is the close proximity of AAM flights to urban environments, which are low in the atmospheric boundary layer (ABL). Continuous and discrete turbulence models based on the well-known Dryden and von Karman spectra^8^ set disturbance magnitude for open-air flight. Specifications of these models provide turbulence intensity and length scales in great detail for medium- to high-altitude operating conditions and have a long lineage of usage in military^9^ and civil aviation applications^10^. Additionally, low-altitude models used are simply modified versions of the high-altitude models with different turbulence length scales, magnitude of inputs, and a reduction in vertical velocity component owing to the presence of the surface. Two primary deficiencies of existing low-altitude turbulence models are apparent for low-altitude AAM applications: (1) The existing turbulence models are built on the assumption of isotropic turbulent flow^11^, which is not appropriate for low-altitudes, and (2) The continuous turbulence models have no capability to model the hazards imposed by bulk flow disturbances (like buildings) found in dense urban areas^12^.

On the contrary, employing high-fidelity models, such as those rooted in computational fluid dynamics (CFD), poses computational challenges, rendering them impractical for AAM operations that necessitate real-time predictions. While CFD-simulated data holds potential for crafting robust algorithms^13^, a more pragmatic approach is to alleviate computational demands by developing surrogate models or Reduced Order Models (ROMs)^14^. ROMs are designed to swiftly approximate numerical simulations, ensuring satisfactory preservation of simulation accuracy^15,16^. These models can adopt a non-intrusive stance^17^ and might incorporate contemporary methodologies, including Machine Learning, either to produce realistic wind data^18–20^ or to deliver predictions^21,22^. Given that Proper Orthogonal Decomposition (POD) underpins the majority of non-intrusive reduced order models^23,24^, it was selected for generating the reduced order wind field in this study.

The main contribution of this paper is to present accurate models of wind conditions and aeromechanics of an AAM vehicle in close proximity to a large building, via one-way coupling with various levels of information present. As per the authors’ knowledge, no such study has been previously performed for a fixed-wing AAM. The model of the vehicle is medium-fidelity, based on solving forces and moments with an Unsteady Vortex Lattice Method (UVLM), appropriate for fixed-wing applications operating in low-speed, low-altitude flight. The operating conditions are characterized by Large Eddy Simulation (LES) and subsequently via reduced order representations of the full LES wind data. Coupling UVLM with LES allows for highly accurate trajectory simulations of the vehicle flying near the structure. Disturbances include time-accurate representation of the wakes and vortical flow structures, some of which are persistent and some transitory. Flow structures resolved are small enough that the full spanwise extent of the aircraft’s wings may not be fully enveloped as the flight progresses through the wind field, and some flow structures are removed through the model reduction process. The effects of temporally and spatially varying realistic wind fields are fully captured by feeding the UVLM-based aerodynamic model with the dynamic wind at each aerodynamic control point and capturing the resulting impact on the trajectory. The results provide insight into the level of fidelity required to simulate passenger-carrying AAM vehicles in urban wind environments and have applications for real-time trajectory planning, model validation, and control design.

## Methodology

This section introduces the governing equations and turbulence closure used for generating the Large Eddy Simulations and the algorithm to generate reduced-order wind data. We also make comparisons to demonstrate the effect of reduced information content on the reconstructed wind. Subsequently, we cover the flight dynamics modeling methodology, emphasizing the aerodynamic force calculation, used to determine the vehicle’s dynamic response.

### Computational fluid dynamics setup

A LES approach is used to resolve the realistic urban wind field. LES data is obtained using Open-source Field Operation and Manipulation (OpenFOAM)^25^. OpenFOAM constitutes a C++ CFD toolbox for customized numerical solvers that can perform simulations of basic CFD, combustion, turbulence modeling, multi-phase flow, stress analysis, and other physical systems^26^. We solve the incompressible Navier-Stokes equations in Boussinesq-approximated form. Details about the governing equations solved and the Sub Grid Scale (SGS) turbulence model used for LES closure are discussed in the sections below.

#### Domain and simulation setup

We defined a computational domain around a prominent, isolated structure for our simulation. Specifically, we chose Boone-Pickens Stadium, positioned at a latitude of 36°7′32.5″ N and a longitude of 97°4′1.7″ W, on the Oklahoma State University campus in Stillwater, Oklahoma, USA. In the domain, the Cartesian coordinate axes, denoted as $x$, $y$, and $z$, align with the geographical orientations of east, north, and upward, respectively. The wind is modeled to flow from the west to the east and the wind profile at the inlet is taken from the outlet face of a precursor RANS simulation. The precursor simulation is run based on the recommendations in^27^ adjusting the domain used in the work such that its outlet face matches the inlet of the current simulation domain while retaining similar grid spacing. Following^27^, a domain of length 5 km is chosen with inlet wind profile specified using log-law with its value corresponding to 8 m/s at reference height ($z_{ref}$) 50 m and roughness length ($z_0$) 0.33^28^. OpenFOAM’s atmBoundaryLayer boundary condition is used to generate the inlet profile for the precursor and atmNutkWallFunction as wall function to account for roughness in atmospheric boundary layer modeling. Divergence Free Synthetic Eddy Method (DF-SEM)^29^ was used to generate turbulence at the inlet of the simulation. Reynolds stress tensor, integral length scales, and velocity profile were interpolated from the precursor simulation. OpenFOAM files to reproduce both the precursor and simulation have been provided at https://osf.io/gucdm/, the data repository corresponding to this work.

**Figure 1:**
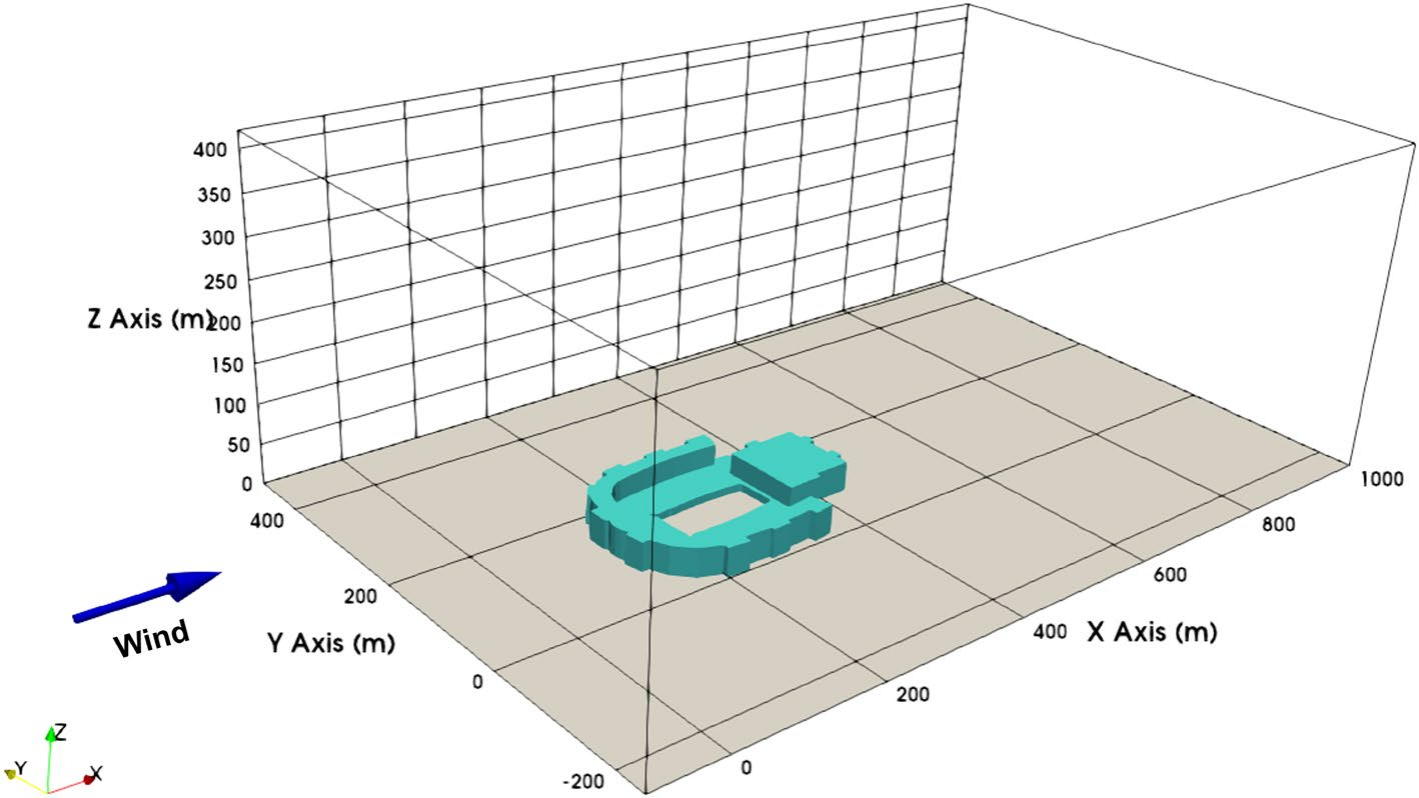
Domain setup and wind direction relative to the building (axis measurements in meters).

For a deeper look into the domain we used for the simulation, please refer to Fig. 1 and Table 1. The domain size was chosen to satisfy the requirements of at least 3% blockage ratio, 5H height above the building, 3H width from the building on one side of the domain, 5H length upstream of the building, and 15H downstream (where $H=40 \, \text {m}$) as recommended by Franke et al.^30^. We constructed the grid using a background mesh resolution of $10 \, \text {m}$ by employing the snappyHexMesh tool available in OpenFOAM. We employed a grid refinement box to optimize the mesh around the structure. Additionally, surface refinement was activated to produce a body-fitted mesh. Comprehensive mesh details, including element types and domain specifications, can be found in Table 1.

**Table 1:** Computational domain and mesh information.

Mesh statistics	
Length of domain in x-dir	1100 m
Length of domain in y-dir	720 m
Length of domain in z-dir	420 m
Background mesh	110 × 72 × 42
Refinement box min.	(− 100 m, − 50 m, 0 m)
Refinement box max.	(750 m, 350 m, 75 m)
Refinement box level	2
Surface refinement level	2 to 4
Hexahedra	2,043,998
Prisms	10,781
Polyhedra	94,847
Total cells	2,149,916

A wind speed of $8 \, \text {m/s}$ (approximately $18 \, \text {mph}$) has been selected for the simulation since this value is representative of moderate breeze conditions according to the US Department of Commerce^31^. Furthermore, at a height of $50 \, \text {m}$, the windiest regions in Oklahoma exhibit an average wind speed that closely matches this value^32^. Therefore, this scenario presents a reasonably challenging test for AAM aircraft under realistic conditions.

**Figure 2:**
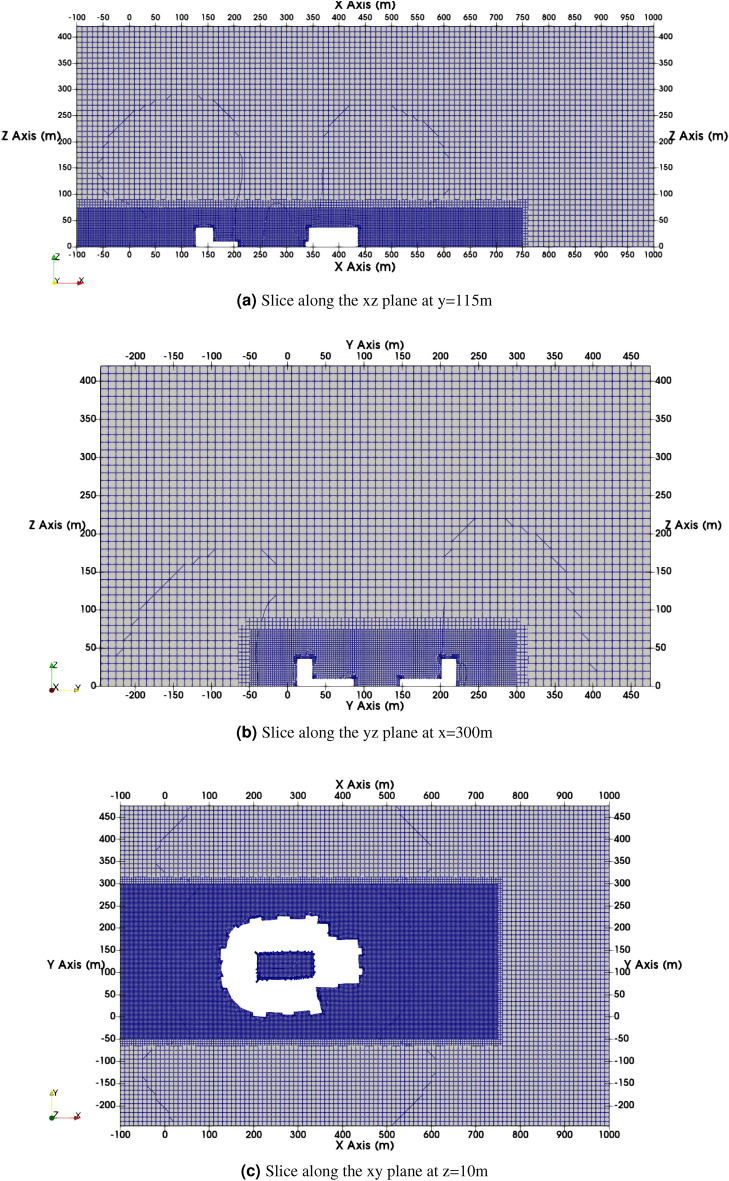
Figures depicting the mesh used for the CFD simulations.

Outlet conditions have been specified for the eastern face, and slip conditions have been specified for the northern, southern, and upward faces. Smooth wall conditions have been applied to the structure within the domain and rough wall conditions(similar to precursor) at its bottom face. The computational mesh chosen for this simulation is depicted in Fig. 2 and is designed to provide sufficient resolution. For generating the LES data, second order implicit scheme for time integration, second order cell limited central scheme for the gradients, and bounded, limited second order upwind for the divergence is used. For more details the reader is referred to the OpenFOAM files provided in the data repository at https://osf.io/gucdm/ and documentation^33^.

In the LES context, attaining a grid-independent solution is inherently challenging. Given our utilization of implicit filtering, the LES Index of Quality, denoted as $LES_{IQ}$^34^, serves as a criterion to ascertain the suitability of a grid. Specifically, it assesses the grid’s capability to resolve turbulent kinetic energy. The $LES_{IQ}$ is computed based on the resolved and total kinetic energies, as shown in Eq. (1).

1 $$\begin{aligned} LES_{IQ} = \frac{k_{res}}{k_{tot}} = \frac{k_{res}}{k_{res}+k_{SGS}+k_{num}} = 1 - \frac{k_{tot}-k_{res}}{k_{tot}} \end{aligned}$$

We can restructure Eq. (1) by recognizing that the total kinetic energy, denoted as $k_{tot}$, is composed of three distinct components: the resolved kinetic energy ($k_{res}$), the contribution stemming from the Sub-Grid Scale (SGS) model ($k_{SGS}$), and the contribution due to numerical dissipation ($k_{num}$). In accordance with Richardson’s extrapolation^35,36^, it is assumed that the combined influence of the SGS model and numerical diffusion scales with grid size, as depicted in (2).

2 $$\begin{aligned} k_{tot} - k_{res,i} = a_k h_i^n \end{aligned}$$

where $n = 2$ denotes the order of accuracy associated with the numerical scheme $h_i$ denotes the average grid spacing for the mesh. The coefficient $a_k$ is determined by solving the two equations (2) from two distinct grids $(i=1,2)$. After determining $a_k$, $LES_{IQ}$ corresponding to the mesh is then calculated using Eq. (3). For further details on computing $LES_{IQ}$, the reader is referred to^34^. In this work, the second mesh is chosen by doubling the number of cells for each direction (effectively halving the grid resolution to 5m) in the background mesh used for snappyHexMesh. As proposed by Pope^37^, an LES computation can be deemed adequately resolved if it captures at least 80% of the turbulent kinetic energy. For the mesh employed in our work, we observed an average $LES_{IQ}$ value > 85%, thereby satisfying the requirement^34^.

3 $$\begin{aligned} LES_{IQ_{i}} = \frac{k_{{res}_{i}}}{k_{{res}_{i}} + \frac{k_{{res}_2} - k_{{res}_{1}}}{\alpha ^n-1} \left( \frac{h_i}{h_2}\right) ^n} \end{aligned}$$

where $\alpha = h_1/h_2 > 1$ and $h_1$ , $h_2$ are the average grid spacing for the meshes. The subscript *i* denotes the mesh under consideration.

#### Governing equations

The governing equations for the flow of an incompressible viscous fluid in Cartesian coordinates are provided by the continuity and momentum equations, which are presented as follows:

4 $$\begin{aligned} \frac{\partial u_i}{\partial x_i}= & {} 0 \end{aligned}$$

5 $$\begin{aligned} \frac{\partial u_i}{\partial t} + \frac{\partial u_{i}u_{j}}{\partial x_j}= & {} - \frac{1}{\rho } \frac{\partial p}{\partial x_i} + \nu \frac{\partial ^2{u_i}}{\partial x_{j} \partial x_{j}} \end{aligned}$$

Upon applying a filter to the aforementioned equations and subsequently simplifying, we derive:

6 $$\begin{aligned} \frac{\partial \overline{u_j}}{\partial x_j}= & {} 0 \end{aligned}$$

7 $$\begin{aligned} \frac{\partial \overline{u_i}}{\partial t} + \frac{\partial \overline{u_i u_j}}{\partial x_j}= & {} -\frac{1}{\rho }\frac{\partial \overline{p}}{\partial x_i} + \nu \frac{\partial ^2{\overline{u_i}}}{\partial {x_j}\partial {x_j}} \end{aligned}$$

However, it is impossible to determine the quantity $\frac{\partial \overline{u_i u_j}}{\partial x_j}$, but the quantity $\frac{\partial \overline{u}_i \overline{u}_j}{\partial x_j}$ is known. We make a substitution and letting $\tau _{ij} = \frac{\partial \overline{u_i u_j}}{\partial x_j} - \frac{\partial \overline{u}_i \overline{u}_j}{\partial x_j}$ results in:

8 $$\begin{aligned} \frac{\partial \overline{u_i}}{\partial t} + \overline{u_j} \frac{\partial \overline{u_i}}{\partial x_j} = -\frac{1}{\rho }\frac{\partial \overline{p}}{\partial x_i} + \nu \frac{\partial ^2{\overline{u_i}}}{\partial {x_j}\partial {x_j}} - \frac{\partial \tau _{ij}}{\partial x_j} \end{aligned}$$

where $u_i$ (with $i = 1, 2, 3$) denotes the velocity components, and $\tau _{ij}$ represents the sub-grid scale stress tensor.

Incorporating the Boussinesq hypothesis, the sub-grid stress can be expressed as:

9 $$\begin{aligned} \tau _{ij} = -2 \nu _t \overline{S}_{ij} + \frac{1}{3} \tau _{kk} \delta _{ij} \end{aligned}$$

where,

$\nu _{t} = \frac{\mu _{t}}{\rho }$, $\mu _t$ is the sub-grid scale turbulent viscosity coefficient.

$$\overline{S}_{ij} = \frac{1}{2}\left( \frac{\partial \overline{u}_i}{\partial x_j} + \frac{\partial \overline{u}_j}{\partial x_i} \right)$$

#### Turbulence closure

This work uses the Wall-Adaptive Local Eddy-viscosity (WALE) SGS closure model^38^. In contrast to the traditional Smagorinsky SGS model, the WALE model considers the effects of the turbulent wall surface and momentum transfer. The sub-grid scale turbulent viscosity is zero in regions of pure shear flow. This ensures the fidelity of the flow field representation, especially in regions close to the wall with laminar flow characteristics. The expression for the sub-grid scale turbulence viscosity coefficient is given by:

10 $$\begin{aligned} \nu _t = \left( C_w \Delta \right) ^2 \frac{ \left( \overline{S}^d_{ij} \overline{S}^d_{ij} \right) ^{\frac{3}{2}} }{ \left( \overline{S}_{ij} \overline{S}_{ij} \right) ^{\frac{5}{2}} + \left( \overline{S}^d_{ij} \overline{S}^d_{ij} \right) ^{\frac{5}{4}} } \end{aligned}$$

where $C_w = 0.325$ and $\Delta$ is the filter scale determined by the lengths of the element in *x*,*y*,*z* directions and $\overline{S}^d_{ij}$ is computed using the relations below,

11 $$\begin{aligned} \overline{S}^d_{ij} = \frac{1}{2} \left( \overline{g}^2_{ij}+ \overline{g}^2_{ji}\right) - \frac{1}{3} \delta _{ij}\overline{g}^2_{kk} \end{aligned}$$

here,

12 $$\begin{aligned} \overline{g}_{ij} = \frac{\partial \overline{u_i}}{\partial x_j} \end{aligned}$$

### Reduced order wind generation

A reduced-order wind field is constructed using POD^39,40^, based on data acquired from LES simulations. The Singular Value Decomposition (SVD) algorithm is employed to compute singular values in increasing order of magnitude for the data. The Relative Information Content (RIC) serves as a criterion to select the requisite number of modes for formulating the reduced-order model. For $N$ modes, the RIC is evaluated as the proportion of the cumulative sum of the singular values up to the $N$th mode relative to their overall sum, as illustrated in Eq. (13).

**Figure 3:**
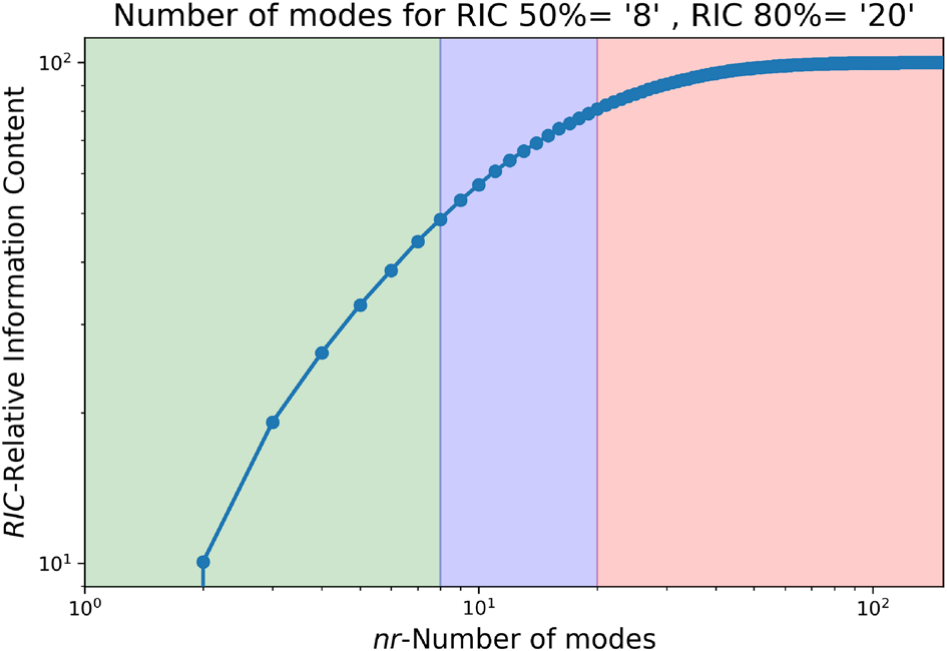
Number of modes for various RIC for u-velocity.

**Figure 4:**
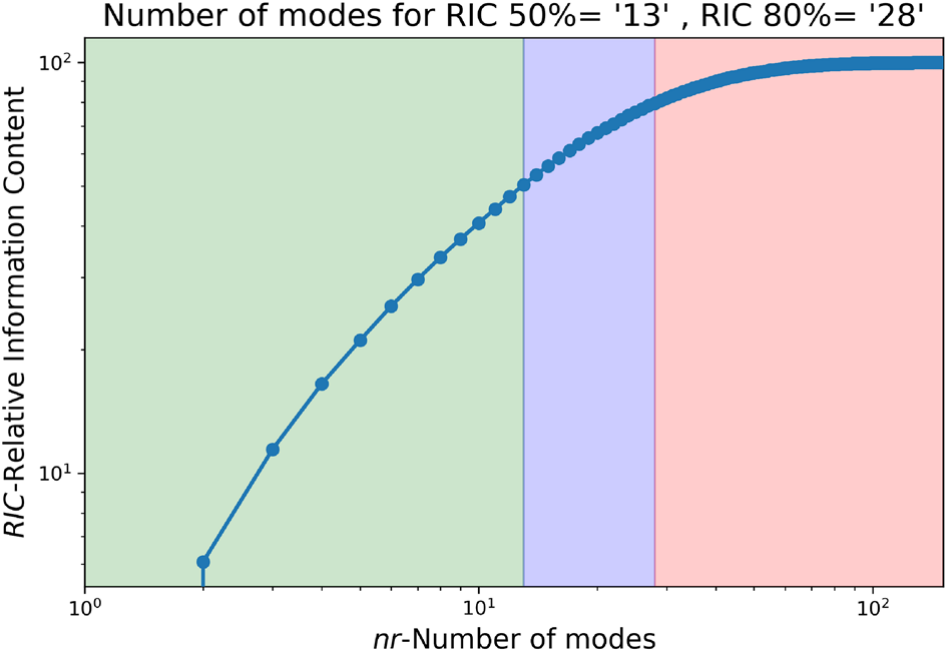
Number of modes for various RIC for v-velocity.

**Figure 5:**
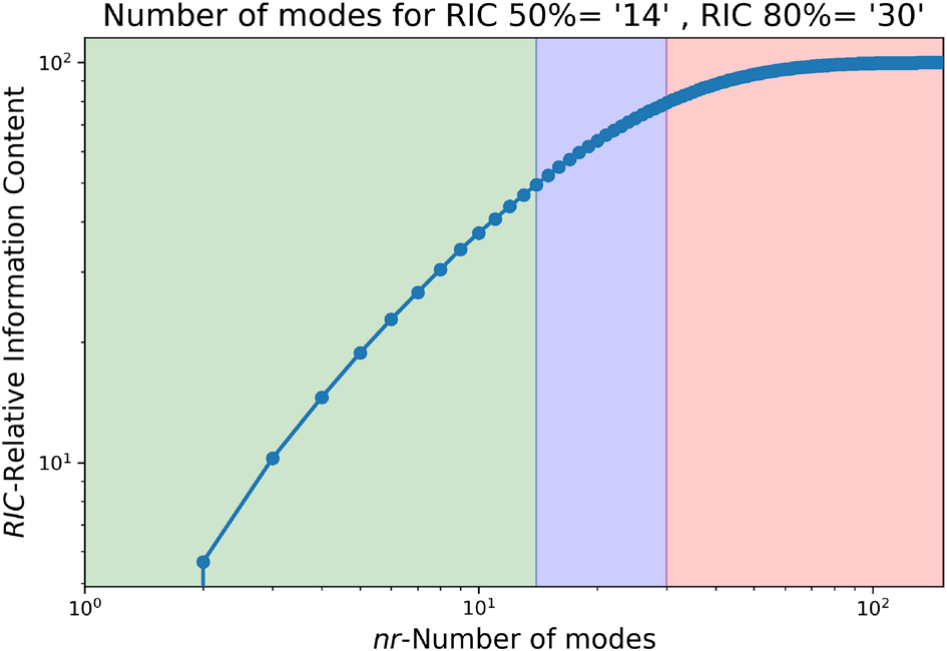
Number of modes for various RIC for w-velocity.

**Figure 6:**
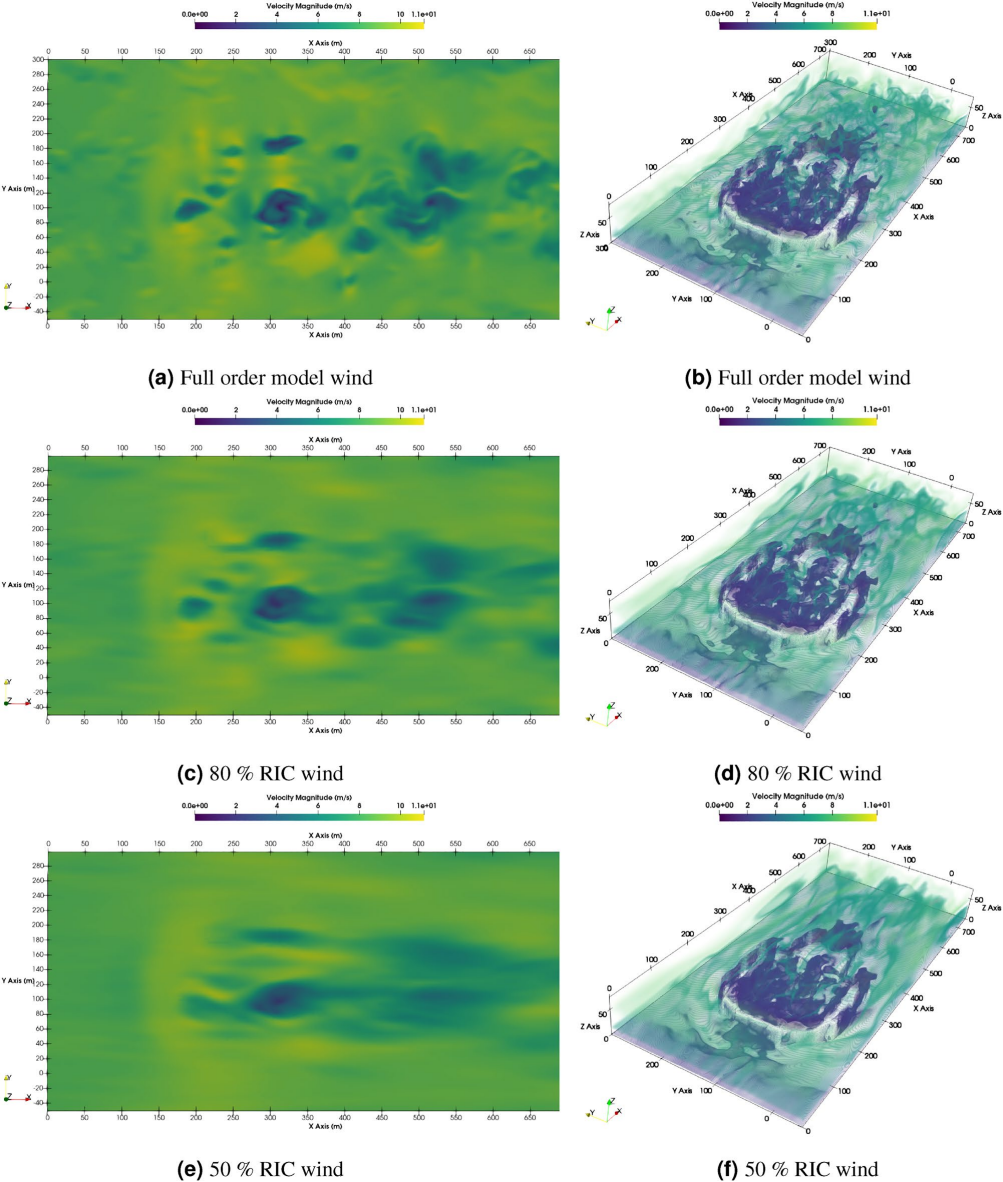
Typical wind velocity magnitude contour at 60 m height and iso-volume comparison of both at 100 s.

The LES data from a smaller domain region spanning from 0 m to 750 m in the x-axis, − 50 m to 300 m in the y-axis, and 0 m to 75 m in the z-axis from the CFD domain shown in Fig. 1 is used for generating the reduced order wind. The LES simulation is run for about 450 s to reach a statistically stationary state. After which the snapshots for 150 s at each 1-s interval are taken for POD. Two different threshold values for RIC of 50% and 80% are selected, and the data for each component of the wind field is decomposed and reconstructed accordingly. Algorithm 1 provides a more detailed explanation of the methodology, and Figs. 3, 4, 5 illustrate the RIC cutoff modes for the velocity components *u*, *v*, and *w*. The impact on the flow field reconstructed using different information content is demonstrated in Fig. 6, where a comparison is made with the original flow field or full-order wind field.

13 $$\begin{aligned} RIC_{N} = \frac{\sum \nolimits _{n=1}^{N}\sigma _n^2}{\sum \nolimits _{n=1}^{N_{tot}}\sigma _n^2} \end{aligned}$$

**Algorithm 1:**
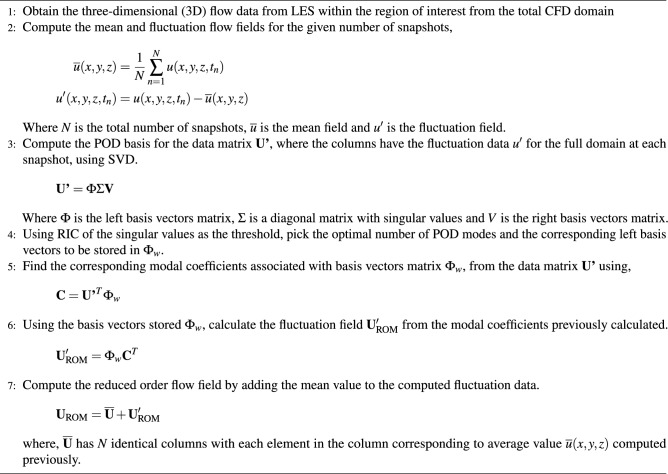
Reduced order wind.

### Flight dynamics modeling

To simulate the response of the aircraft in the 3D turbulent velocity fields, SHARPy^41^ (Simulation of High-Aspect Ration airplanes in Python) will be utilized. SHARPy is an analysis toolbox intended to study the nonlinear aeroelastic behavior of high-aspect-ratio aircraft and wind turbines. The core code is written in Python 3, while more computationally expensive calculations are included in C++ and modern Fortran libraries. A UVLM^42^ computes the aerodynamic forces on all lifting surfaces. The forces developed in the aerodynamic grid are transferred to a geometrically exact composite beam model based on the work from Géradin and Cardona^43^ and Hesse, Palacios, and Murua^44^ to handle structural and rigid body dynamics. SHARPy supports dynamically coupled time marching, which means that structural dynamics, rigid body dynamics, and aerodynamics are all advanced simultaneously. This coordinated advancement is achieved through a Block Gauss-Seidel scheme implemented within each physical time step, ensuring the consistency of fluid-structural interactions^45^.

#### Aerodynamic solver

**Figure 7:**
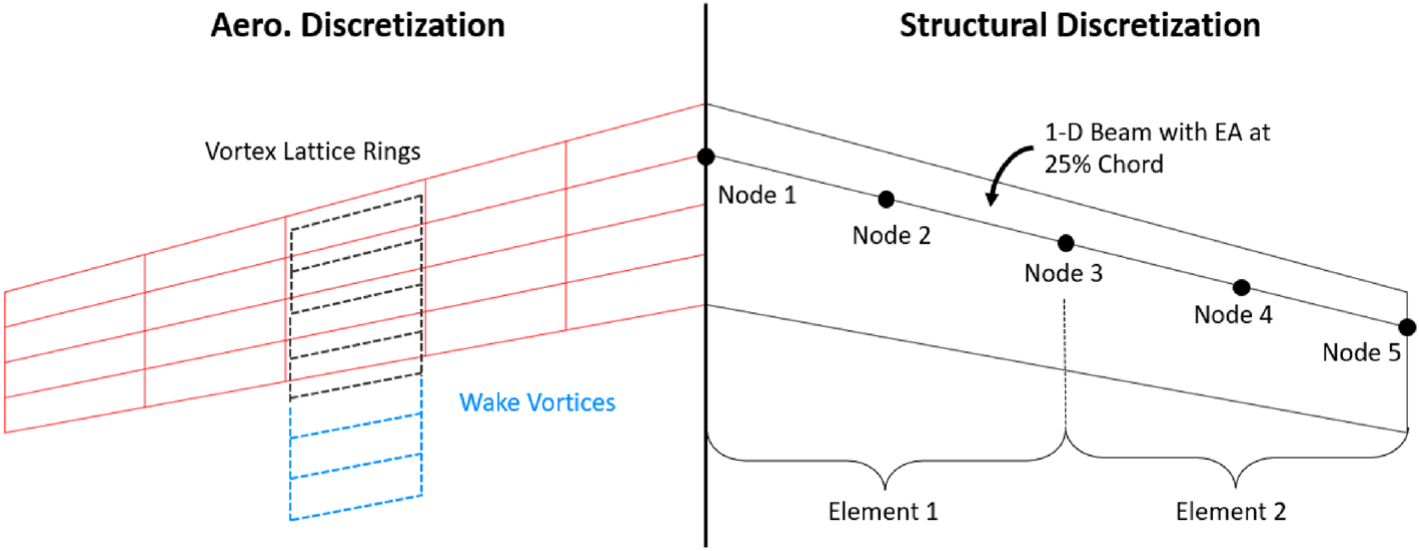
Structural and aerodynamic discretization of wing.

For completeness, additional details describing the SHARPy aerodynamic force calculation are included herein to highlight the way time and spatially varying gust components are included in the aerodynamic force calculation. The work in this paper assumes a rigid aircraft with no structural deformations. The UVLM aerodynamic solver is based on the 3D potential flow theory. Aerodynamic surfaces are represented by distributing rectilinear vortex rings on the camber lines of the various airfoil cross sections that make up the lifting surfaces, as seen on the left wing of Fig. 7. The circulation strength around each of the vortex rings is determined by solving the boundary condition, Eq. (14), at each instance in the simulation by setting $\Gamma _b$ and $\Gamma _w$ such that no velocity is flowing through the vortex rings at the collocation points.

In the dynamic simulation, the circulation strength vector ($\Gamma$) of each vortex ring on the lifting surfaces is computed at each instance by applying the non-penetrative boundary condition simultaneously at the collocation points. The velocity contributions in the boundary condition equation at each collocation point are derived from induced velocities projected by each bound and wake vortex ring panel, oriented normally to the respective collocation point. These contributions are captured by the bound aerodynamic influence coefficients (AIC) matrix ($\mathscr {A}$) and the wake AIC matrix ($\mathscr {A}_w$). Additionally, all other velocity sources ($v_k$) are considered, which include velocities arising from aircraft motion and the turbulent gusts from the LES. The entries in the $\mathscr {A}$ and $\mathscr {A}_w$ matrices are determined using the Biot-Savart law and depend on the panel elements’ geometry at each step in the simulation.

14 $$\begin{aligned} \mathscr {A}\Gamma + \mathscr {A}_w\Gamma _w + v_k = 0 \end{aligned}$$

Within SHARPy, the aerodynamic forces are found using the unsteady form of the Kutta-Joukowski theorem^46^. For completeness, the steady and unsteady force contributions are shown below in Eqs. (15) and (16) considering a single vortex ring. The steady contribution on vortex ring *k* depends on the air density, the net velocity at the midpoint of the vortex segment denoted $v_k$, the segment length $l_k$, and the net circulation $\Gamma _k$. The unsteady force contribution on each bound vortex ring is computed considering the area enclosed by each vortex ring $A_i$, $\hat{n}$ the normal vector, and the time derivative of bound circulation $\dot{\Gamma _k}$.

15 $$\begin{aligned} \partial F_{st}= & {} \rho _{\infty }\Gamma (v \times \delta l) \Rightarrow f_{st} = \rho _{\infty } v_k l_k \Gamma _k, \, k \, \epsilon \, \{0,..., 3\} \end{aligned}$$

16 $$\begin{aligned} \partial F_{unst}= & {} \rho \frac{\partial \Gamma }{\delta t} \Rightarrow f_{unst} = \rho _{\infty } A_k \hat{n_k} \dot{\Gamma _k} \end{aligned}$$

Total aerodynamic force comes from summing the force components at each bound vortex ring. In SHARPy, the aerodynamic forces are mapped to the structural model. For convenience, the structural nodes are aligned with the spanwise discretization of the aerodynamic grid to eliminate the need for interpolation in the force and moment mapping^47^. For this paper, even without structural flexibility effects considered, this methodology is still beneficial as it maintains the spatial variation of aerodynamic forces seen due to wind magnitude variation across the extent of lifting surfaces. Rigid body motion equations are integrated with the elastic degrees of freedom by including the system with translational and angular velocity terms and attitude tracking in the form of quaternions^45^. Integration of the origin is then performed to progress the configuration through time.

The wake panels inherit vorticity from the trailing bound vortex segments of each aerodynamic surface in accordance with the Kelvin condition. Wake panels are maintained until a user-specified distance downstream has been reached where the wake is no longer tracked in order to keep computational costs reasonable. This paper’s specified distance downstream is 8 chord lengths from each aerodynamic surface. The time step for the dynamic, time marching simulation is found using Eq. (17), where each time step corresponds with the shedding of one full wake panel. To calculate this distance, the chord (*c*) is divided by the number of chord-wise panels (*m*) and the trim velocity ($u_\infty$). This keeps the equations of motion in lock-step with the aerodynamic force calculation and helps with convergence within the dynamic coupled time marching solution evaluation. In general, UVLM allows for the full force-free development of the wake by propagating wake panel corner points with the local velocity. This paper uses the convected-background-flow wake model in SHARPy, whereby the wake vorticity convects back in a fixed wake plane. Effects due to wake roll-up in a full force-free wake are ignored, meaning the velocity at each wake corner point does not need to be computed to propagate the wake from one time step to the next. The convected wake model results in essentially equivalent rigid body trajectories compared to the full force-free wake model while saving significant computational effort^11,48^.

17 $$\begin{aligned} dt = \frac{c}{mu_{\infty }} \end{aligned}$$

To introduce external wind fields, HDF5^49^ files containing the grid domain location and the *u*, *v*, and *w* wind velocities in the inertial frame are provided to SHARPy. The wind field is stored at 1-s intervals. Time interpolation between adjacent fields is performed as the time-marching simulation evolves. At the same time, spatial interpolation is used to determine the external gust magnitude at each collocation point of the aerodynamic grid. The $\Gamma$ distribution and aerodynamics force calculation are therefore impacted accordingly. Traditional turbulence models apply velocity perturbations at a single location, most often corresponding to the center of gravity (C.G.). In order to align more closely with traditional turbulence model applications, a feature has been developed to assess gust magnitudes at a single reference point and subsequently apply these magnitudes uniformly across all panels. This capability, referred to herein as the C.G. cases, is used to compare the response of the aircraft when embedded in the full-order model of the wind field to assess the need for spatial interpolation in the presence of non-isotropic flows around large obstructions.

### Aircraft model

**Figure 8:**
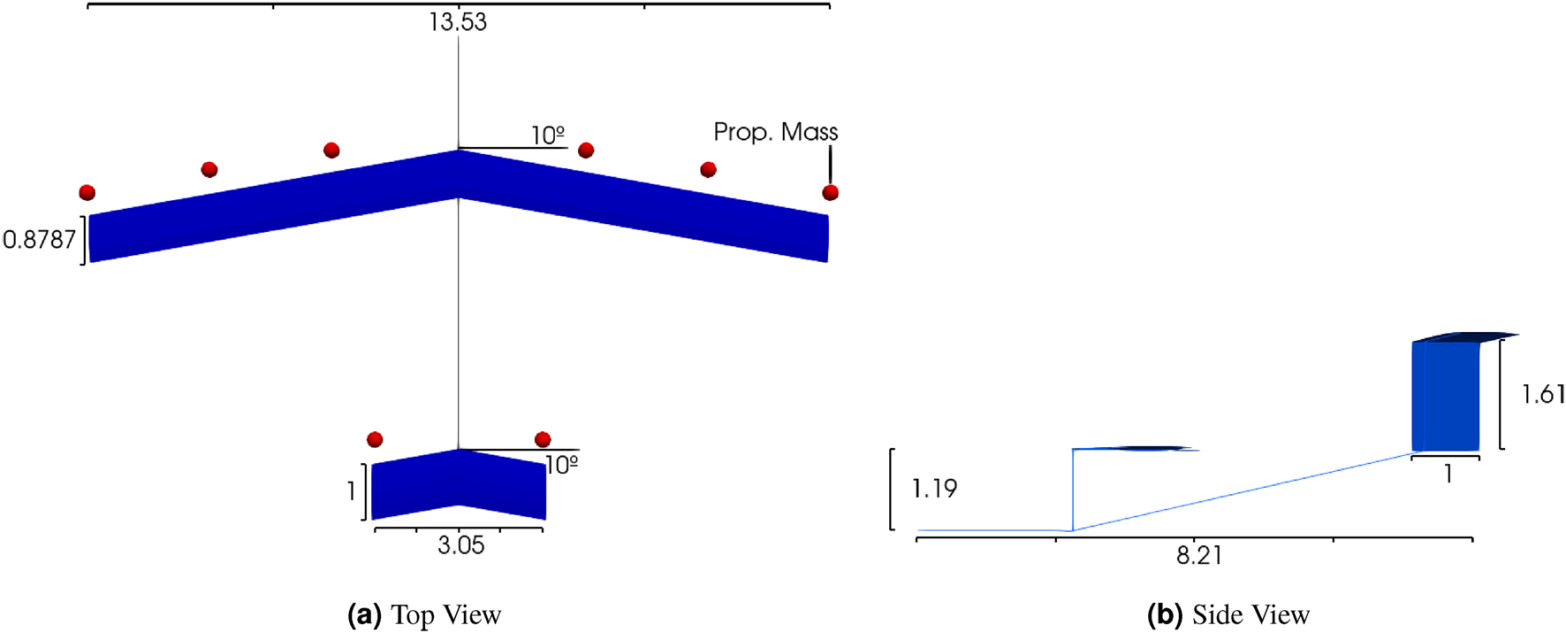
Representative passenger carrying AAM vehicle.

The vehicle model considered in this work is similar to the passenger-carrying tilt-wing AAM concept outlined by NASA^50,51^. The wing and tail planforms, airfoil profiles, weight, inertia distribution, and the overall mass and moments of inertia have been represented in the SHARPy input file to align with the specifications provided in the references. The aircraft’s geometry is visually depicted in Fig. 8, with geometric and mass properties specified in Table 2. A uniform mass per unit length is assumed along the wing, tail surfaces, and the fuselage beam element. The structural mass is distributed appropriately amongst the other mass contributions of the main aircraft components. Lumped masses, representative of the electric motors and propeller blades, are placed at the locations shown in Fig. 8 as called for by the design. All other mass contributions are considered miscellaneous and are consolidated into a single lumped mass element that is placed on the longitudinal axis where needed to achieve a 10% static margin. The mass moments of inertia given in Table 2 are found based on the mass distribution assumptions previously discussed. Mass moment of inertia values were not specified in the NASA reference^50^.

**Table 2:** Properties of the AAM vehicle.

Property	Value
Wing and H-tail airfoil	GA(W)-1 Mod
Vertical tail airfoil	NACA 0020
Structural mass	920.79 kg
Main wing mass	205.47 kg
H-tail mass	63.96 kg
Propulsion mass	1116.74 kg
Misc. mass	654.54 kg
Total mass	2961.5 kg
$I_{xx}$	30,210 kg m^2^
$I_{yy}$	18,460 kg m^2^
$I_{zz}$	44,050 kg m^2^

The concept aircraft designers specify a cruising speed of 79.6 m/s^50^. Simulations in this paper focusing on the encounter with the turbulent velocity field near the stadium are performed for an aircraft trim speed of 67.3 m/s, corresponding to a coefficient of lift ($C_L$) of 0.8. Compared to the normal cruising velocity, this slightly reduced speed is chosen to replicate a scenario where the aircraft is approaching a landing zone and is slowing down in preparation for the transition to vertical flight. Initially, open-loop response characteristics were desired for this investigation. The limited vertical height of the LES computational grid often resulted in the aircraft model departing the top of the domain due to the deflected flow on the windward side of the stadium. Simple proportional feedback control laws were added to compensate for the errors of pitch attitude and roll attitude off of trim. Additionally, some elevator command was added to lightly compensate for altitude error, though most control action is due to attitude maintenance, similar to guidance provided to pilots in turbulent conditions. Feedback control gains held constant for all simulation runs are shown in Table 3.

**Table 3:** Feedback control gains.

Control channel	Proportional
Aileron to roll attitude	1.5
Elevator to pitch attitude	2.0
Elevator to altitude error	0.015

**Figure 9:**
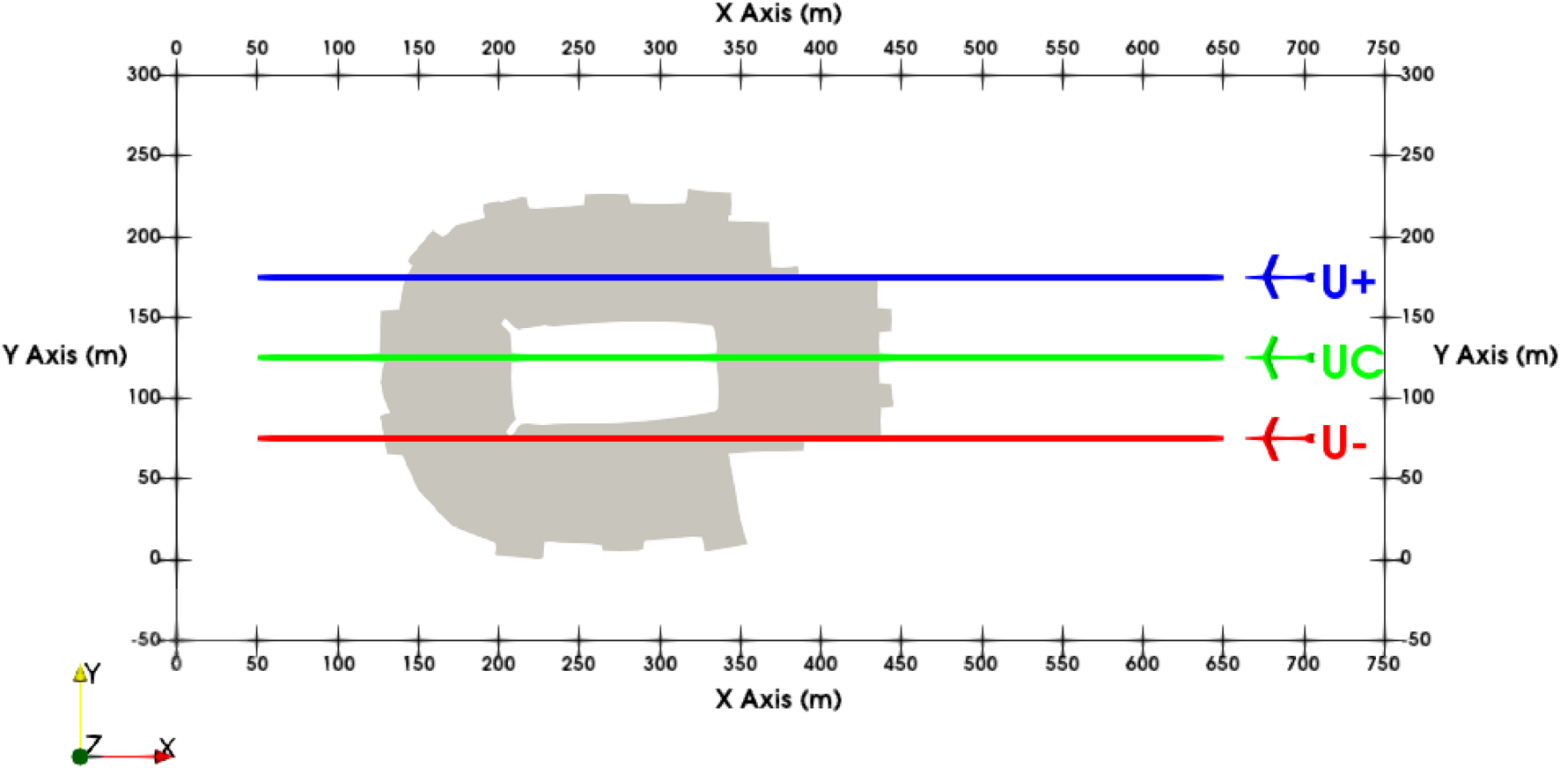
Starting positions and nominal path of the aircraft relative to the stadium at an initial altitude of 60 m.

## Results and discussion

This section presents simulation results for the conceptual AAM vehicle upon encountering the turbulent wind field around the stadium. Three different starting locations, Fig. 9, are used to obtain the results for this paper. The first position was selected at the center of the stadium as relative wind magnitudes and changes in wind structures appeared to be the greatest at this location. This position is denoted as UC (upstream, center). Additional locations offset from the UC position by ± 50 m (U+, U−) are also evaluated further to explore the configuration’s response within the wind field. All cases begin at a height of 60 m which is 20 m above the height of the building.

First, results are presented that highlight the impact of computing spatially varying aerodynamic loads in the time-varying realistic wind field. Next, the impact of model order reduction on the LES data is highlighted. For both cases, the 95% confidence intervals on the trajectories are used to identify significant differences in the response. These confidence intervals are found using Student’s T-statistic, summarized by Eq. (18)^52^, where $\sigma$ is the standard deviation, and *n* is the number of independent samples in the data set. The trajectory data points were grouped by time step in the development of the confidence intervals. All results can be accessed at the reader’s convenience at the shared repository listed in the Data Availability Statement.

18 $$\begin{aligned} CI ={\bar{y}} \pm t_{\alpha /2, \ df}\ \frac{\sigma }{\sqrt{n}} \end{aligned}$$

### CG vs spatial in full order model wind

**Figure 10:**
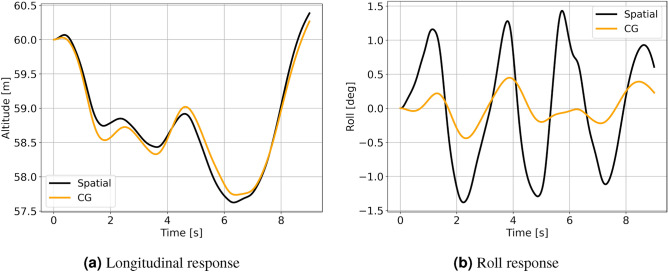
UC sample gust response between spatial variation and CG cases in FOM wind.

**Figure 11:**
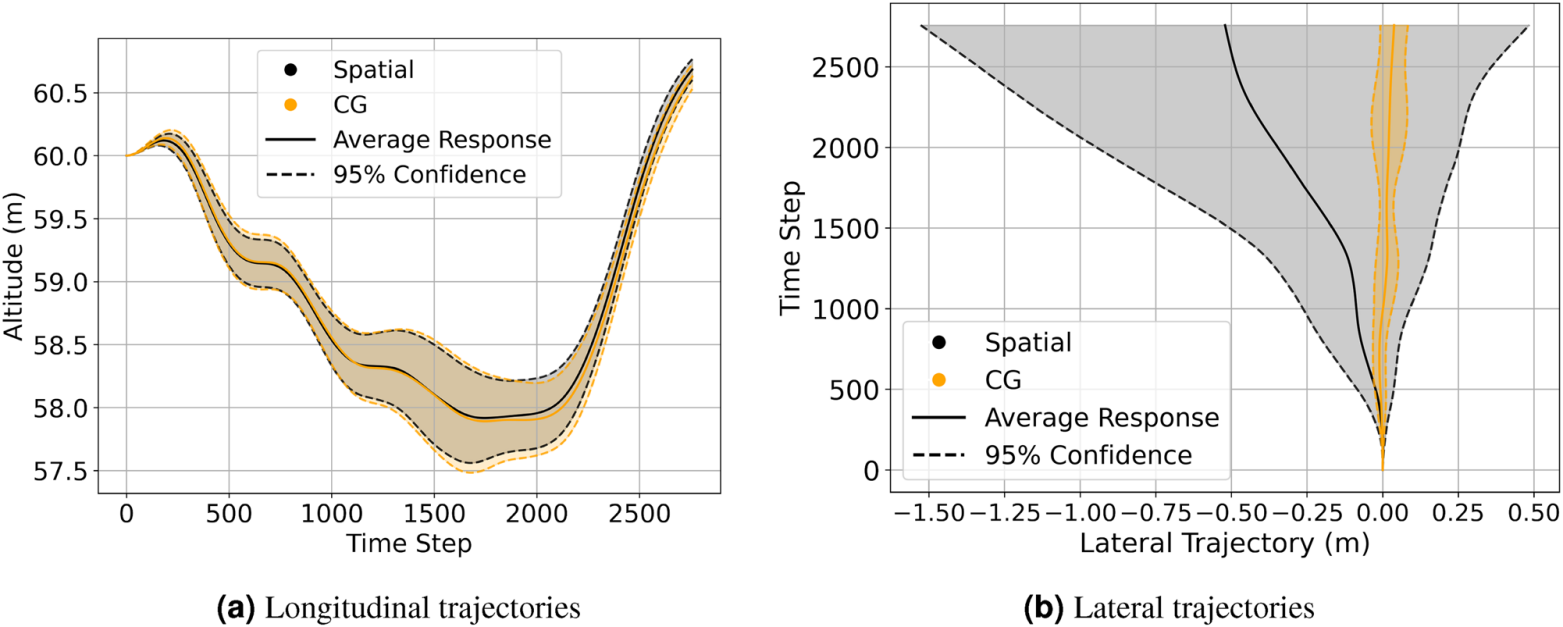
UC Trajectories with 95% confidence interval between the spatially varying and CG cases.

The comparison between the full spatially varying, designated “Spatial,” and the single reference point (CG) cases are both evaluated using the full-order wind field at the UC position. To reiterate, the Spatial cases use spatial interpolation to determine the gust magnitudes at each collocation point in the aerodynamic grid, while the CG cases apply the gust magnitudes only found at the center of gravity. 15 runs were obtained for different initial start times in the 150 s of the LES wind field, with each simulation representing 9 s of flight time. Each initial time step is separated by 10 s relative to the wind field so that a variety of different wind structures are encountered. A representative run for both cases in the longitudinal and lateral directions is given in Fig. 10. The average longitudinal and lateral trajectories and the associated 95% confidence intervals between the two cases are shown in Fig. 11.

### Full order model wind, 80% ROM, 50% ROM and mean wind comparison

**Figure 12:**
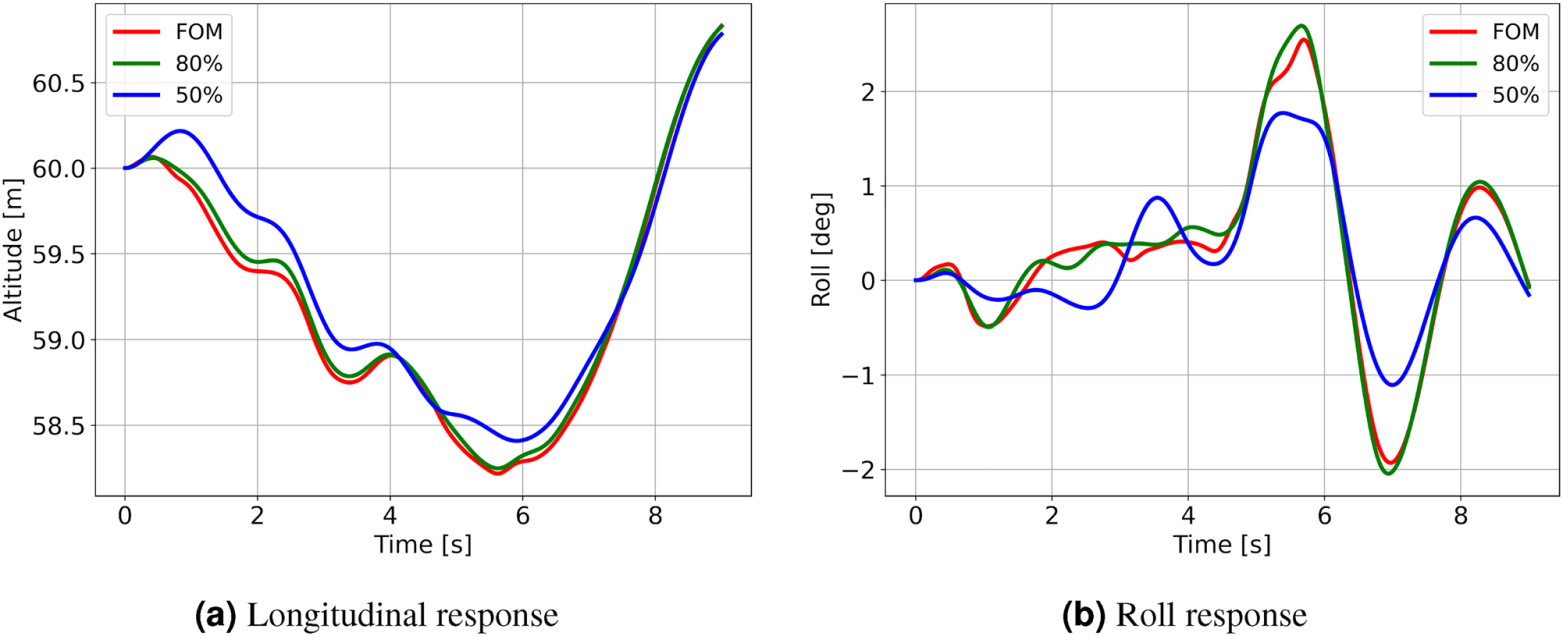
U+ sample gust response between FOM and ROM cases.

**Figure 13:**
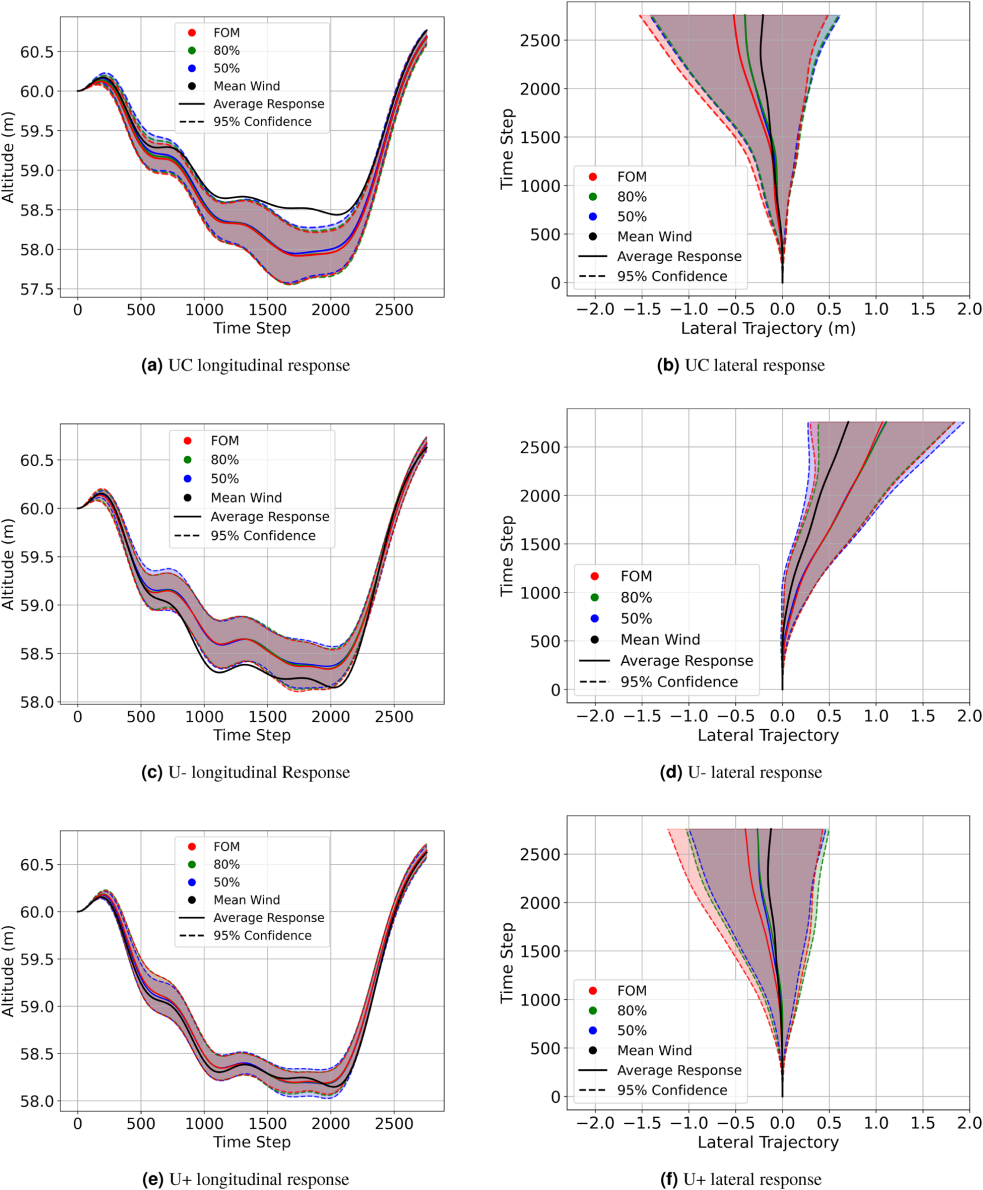
Trajectories with 95% confidence interval.

To compare the effect of different orders of wind field (FOM, 80% ROM, and 50% ROM) on the vehicle dynamics statistically, 15 runs were obtained, each starting at positions (UC, U+, U−). The start time for each run was again varied at an interval of 10 s each so that as many different wind structures were encountered as possible. The longitudinal and roll response for a representative case from this set of runs is depicted in Fig. 12. 95% confidence intervals for the trajectories at starting locations (UC, U+, U−) are shown in Fig. 13. In addition to the ROM, the mean wind field trajectories are also presented. The mean wind field is a temporal average of the velocity field from LES at all the locations on the mesh.

Table 4 presents the standard deviation of all control surface activity for the ailerons and elevators. The standard deviation of control deflection over a maneuver time history is a metric of control activity over a maneuver^53^ and enables further comparison between the full-order and reduced-order wind models.

**Table 4:** Standard deviations of the control surface deflections.

Wind field	UC	U+	U−
FOM ail	1.46	1.10	1.39
80% ail	1.53	1.11	1.38
50% ail	1.40	1.01	1.37
Mean wind ail	0.25	0.16	0.26
FOM elv	1.45	1.43	1.51
80% elv	1.46	1.45	1.50
50% elv	1.46	1.41	1.46
Mean wind elv	1.31	1.37	1.35

## Conclusions

Considering the comparison between the spatially varying wind versus the wind applied only at the CG (Figs. 10 and 11), the largest differences are seen in the lateral response trajectories (Fig. 11b). The wingspan of the aircraft is larger than the length scale of the finer structures that are resolved in the LES simulations, so the spatially varying wind model captures asymmetric loading on the aerodynamic surfaces with spanwise variation that is clearly not seen in the CG cases, as evidenced by the activity in the roll response and relative width of the confidence intervals. This also shows that asymmetric loading of the planform in the vertical (z) and longitudinal (x) wind directions is greater than the presence of lateral (y) wind gusts in inducing roll responses for these sets of results.

The longitudinal trajectories reveal minimal differences between the CG and the spatially varying wind scenarios. This finding implies that the entirety of the lifting surfaces confronts the horizontal gust velocity variations almost simultaneously, consistent with the primary flow being a direct headwind. The confidence intervals in Fig. 11a exhibit only minor disparities. This underscores the efficacy of the CG approach in capturing the longitudinal response when the predominant wind aligns with the flight direction.

The longitudinal trajectory responses remain consistent whether subjected to the FOM, ROM, or the mean wind field, Fig. 13a,c,e. The most pronounced distinctions are evident in the lateral trajectories (Fig. 13b,d,f), though these differences are still minor. Reduced fidelity in the ROM’s, is likely the reason for these disparities due to the resulting changes in the asymmetric loading of the planform. Table 4 shows the relationships are not straightforward as the control activity trends appear to be case-specific. This is not unexpected as the changes in the asymmetric loading would not vary in a predictable way as the loss of fidelity could lead to both an increase or decrease in the overall roll moment experienced depending on the wind structures. It is clear though, that the mean wind responses are considerably different when compared to the responses of the FOM and ROMs. This is evidenced by the longitudinal trajectories of the mean wind exceeding the 95% confidence intervals in both the UC and U− cases. Additionally, the control activity of all mean wind cases is less than their FOM and ROM’s equivalents as shown in Table 4, of which the aileron control activity is of concern as it is greatly under-predicted. The results strongly suggest that a temporally- and spatially-varying wind disturbance is required to adequately represent the flight dynamics and control usage required to navigate the disturbances caused by operating near large structures like buildings. This observation justifies the use of LES simulations to develop the wind field data, as opposed to an approach like Reynolds-averaged Navier-Stokes (RANS). The reduced order representations of the wind field do not have a significant impact on the resulting flight dynamics in this case.

Numerous observations articulated within this study are contingent on the high wing loading of the AAM aircraft configuration, and the results are exclusively obtained from upwind direction scenarios. An AAM configuration characterized by lower wing loading is anticipated to yield larger differences in trajectory, greatly expanding the confidence intervals. Furthermore, the influence of spatial variation is reliant on the comparative size of wind structures to the aircraft configuration. These considerations warrant careful attention when integrating robustness into attitude-hold controllers and leave much room for future work considering different vehicle configurations, and environments more representative of a full urban cityscape.

## Data Availability

The data supporting this study’s findings and additional details to replicate are openly available in the “Modeling Realistic Urban Winds for Advanced Air Mobility Aircraft” repository on Open Science Framework at https://doi.org/10.17605/osf.io/gucdm.
